# Effects of blastocyst elongation and implantation chamber formation on the alignment of the embryonic axis and uterine axis in mice

**DOI:** 10.3389/fcell.2024.1421222

**Published:** 2024-06-14

**Authors:** Jun Sakurai, Sanae Oka, Yoko Higuchi, Sonoko Ohsawa, Toshihiko Fujimori

**Affiliations:** ^1^ Division of Embryology, National Institute for Basic Biology, Okazaki, Japan; ^2^ Deapartment of Basic Biology, School of Life Science, The Graduate University for Advanced Studies, SOKENDAI, Okazaki, Japan; ^3^ Model Organisms Facility, Trans-Scale Biology Center, National Institute for Basic Biology, Okazaki, Japan

**Keywords:** embryonic implantation, blastocyst, embryonic axis, uterine axis, implantation chamber

## Abstract

Embryo implantation involves a series of events that bring the embryo and maternal tissues into contact to support post-implantation development in mammals. During implantation, alignment of the embryonic–abembryonic (E–Ab) axis of the blastocyst with the mesometrial–antimesometrial (M–AM) axis of the uterus precedes post-implantation embryonic development and placentation. In the present study, we observed the morphological changes in blastocysts and the endometrial luminal epithelium (LE) that occur during the alignment of the embryonic and the uterine axes. We found that at the time that the blastocysts attached to the LE at the mural trophectoderm, the embryonic axis was not aligned with the uterine axis. Alignment of the embryonic E–Ab axis with the uterine M–AM axis occurred after E4.0, and the embryo was significantly elongated during the process. The depth of the implantation chamber (IC) correlated with the degree of alignment, suggesting that elongated embryos are oriented along the M–AM axis during IC formation. Transplantation of the Concanavalin A (Con A)–coated beads induced IC formation, and the alignment of two Con A–coated beads present in the same IC in the M–AM direction suggested that elongated materials can align along the M–AM axis. These data suggest that an elongated shape of the embryo and IC formation coordinate the alignment of the embryonic and uterine axes.

## Introduction

Embryo implantation, which is the first stage of gestation in mammals, is the process by which direct interaction between the embryo and uterine tissues is established in the pregnant female (Cha et al., 2012; Chen et al., 2013; Cha and Dey, 2014). In mice, embryos at the blastocyst stage are transported from the oviduct and enter the uterus 3 days after fertilization. The blastocysts adopt a position in which adequate distance between neighboring blastocysts in the uterus is achieved by myometrial contractions and LPA3 signaling (Pusey et al., 1980; Ye et al., 2005; Hama et al., 2007; Chen et al., 2011; Flores et al., 2020). Blastocysts that hatch from the zona pellucida are ready to attach to the endometrial luminal epithelium (LE) and implant in the precise orientation. The blastocyst consists of 2 cell types, the inner cell mass (ICM) and the trophectoderm (TE); the ICM is located on one side of the blastocyst, which represents the axis of the embryo and is known as the embryonic–abembryonic (E–Ab) axis (Kurotaki et al., 2007; Saiz and Plusa, 2013).

In rodents, the bicornuate uterus consists of two uterine horns on the left and right sides. The uterine arteries are located on the mesometrium parallel to the extending uterine horns longitudinally and attached to one side of the uterus (Liebgott, 1984). In a transverse section of the uterus, the mesometrium and main uterine arteries protrude at one end of the circular tissue. Thus, the uterus is a polarized tissue with two defined axes, the longitudinal axis and the mesometrial to anti-mesometrial (M–AM) axis.

After implantation, the blastocysts localize to the anti-mesometrial side of the uterine lumen, which is lined with the LE, and the embryonic side of the blastocysts is located on the mesometrial side. The E–Ab axis of the embryo is aligned with the uterine M–AM axis (Kirby et al., 1967; Wimsatt, 1975; Chen et al., 2013). The orientation of the embryo relative to the uterus is important for further post-implantation embryonic development because the position of the future placenta is determined at this stage. In the later stages of pregnancy, the placenta forms between the embryo and the mesometrium, which provides vascular connections to the placenta. Misalignment of the placenta in the mouse uterus affects embryonic development (Wilkinson et al., 2021). The placenta is derived from the TE covering ICM of the blastocyst in rodents and it forms on the mesometrial side where the vasculature connects it to the uterus for the exchange of oxygen and nutrients between the mother and the fetus.

This correlation between the orientation of the embryo and the positioning of the placenta is not limited to rodents, and occurs in many other species although there are differences in the attachment of the implanting blastocyst among species. In rodents that undergo interstitial attachment, the blastocysts attach to the LE at the mural TE, which induces a decidual reaction in the surrounding stromal cells (Chakraborty et al., 1996; Lim et al., 1997; Paria et al., 2000; Massimiani et al., 2019). This triggers cell death at the LE, followed by invasion of embryonic cells into the inner part of the endometrium (Li et al., 2015). In cattle and sheep, the mural TE is in contact with the LE, and this contact is maintained during embryogenesis, which is called superficial attachment (Chavatte-Palmer and Guillomot, 2007; Valadão et al., 2019). In primates and horse, the blastocyst attaches to the LE with the polar TE (Salamonsen, 1999; Silva and Ginther, 2006; Muter et al., 2023). These species share certain features regarding the orientation of the embryo during implantation to determine the position of the future placenta relative to the mesometrium in the uterus (Rasweiler and Badwaik, 1999; Qi et al., 2009). Hypothetical models have been reported in rodents (Kirby et al., 1967), and recent findings indicate that abnormal uterine folds affect the orientation of the blastocyst during implantation (Zhang et al., 2014; Madhavan et al., 2022). However, the detailed mechanism and significance of the alignment and orientation of the embryo during implantation remain to be clarified.

The aim of this study is to elucidate the mechanism underlying the alignment of the embryonic and uterine axes during implantation. For this purpose, we examined the process of embryonic axis alignment in the mouse uterus as well as changes in the morphology and positioning of embryos. We also examined the morphological relationship between the uterus and the embryo during the alignment of their axes in implantation.

## Materials and methods

### Animals

Animals were maintained in a light- and temperature-controlled room with a 12-h light/dark cycle at 23°C ± 2°C. All animal experiments were approved by the Animal Research Committee of National Institutes of Natural Sciences (23A030). Wild-type female mice (slc; ICR, Japan SLC) were used for the analysis. Lif (Leukemia inhibitory factor) deficient mutant mouse lines were established by the CRISPR/Cas9 system in our laboratory. The crRNAs were designed to make deletions to include exons 2 and 3 of the *lif* gene using the web design tool, Konezumi (Kuno et al., 2019) as follows: left crRNA (ATC​CCT​GGA​AGA​GAC​ACG​GA) and right crRNA (GTG​GTC​CAG​GCC​TTC​TAG​AG). The crRNA, tracrRNA, and Cas9 protein (#1081058, IDT) were introduced into fertilized eggs from the c57Bl/6 strain by electroporation [Pulse mode, 30 V; Pd(+), 30 V (3 ms ON, 97 ms OFF) × seven cycles] and transplanted into pseudopregnant females. F0 mice carrying the mutation were selected by PCR and sequencing. To detect the *lif* gene mutation in F1 or later generations, the following primers were used: forward primer (5′-CCA​GGA​GGG​ATG​AGG​CTA​GA-3′) and reverse primer (5′- GCA​GCC​CCG​TTT​TGC​ATT​TA-3′), yielding a PCR product of 1,574 bp in the WT allele and 409 bp in the mutant allele. LATaq or MightyAmp (RR002B or R076B, TaKaRa bio) were used for the genotyping PCR experiments. The Lif deficient mouse line was maintained with the ICR background. Female 10–28-week-old mice were selected randomly and crossed with males to induce pregnancy. The noon of the virginal plug detection was designated as E0.5 (embryonic day 0.5).

### Histology

Whole mouse uteri were dissected from pregnant mice and fixed in 4% paraformaldehyde (PFA) for 2 days at 4°C. After fixation, the tissues were washed three times with PBS, dehydrated in 70%, 90%, and 100% EtOH, immersed in chloroform and 100% paraffin wax (Paraplast plus, Tyco) at 62°C, and embedded in paraffin. For transverse observations, the uteri were cut into 3 mm sections using a razor blade in paraffin and embedded to make the transverse plane the cutting plane. For longitudinal sections, the whole uterine horn was embedded in paraffin with the M-AM axis parallel to the plane of sectioning (Supplementary Figure S1A). Tissue blocks were sectioned at 5 μm using a microtome with flow function (HM340E, Microm), and the sections were placed on slide glasses (S2444, Matsunami). Hematoxylin and eosin staining (No. 3000–2 and No. 32053, Muto) was performed using an automatic staining machine (Leica Autostainer XL Automated Slide Stainer, RRID:SCR_020212), and samples were mounted in MGK-S (FK00500, Matsunami) with coverslips (Matsunami). Serial sections were scanned using a slide scanner (Leica SCN400 slide scanner, RRID:SCR_023611) and observed with a viewer (Aperio ImageScope, RRID:SCR_020993).

### Alignment of serial images and 3D reconstruction

The alignment programs developed in collaboration with Prof. Seiichi Uchida’s group at Kyushu University were used to align the serial images detected by the slide scanner (a paper is under preparation, unpublished). The Regions Of Interest (ROIs) were manually drawn at regular intervals in the images, and similar regions were selected and aligned by identifying key features present in the ROIs of two images (SURF: Speeded Up Robust Features) using alignment programs.

The contiguous tissue images thus identified were saved as tiff files and used to extract the LE using Fiji (RRID:SCR_002285), followed by the construction of three-dimensional (3D) images.

### 3D reconstruction of the blastocyst using serial sections

The regions of uterine tissue including the blastocyst were segmented from the images acquired by a slide scanner using TrackEM2, a Fiji plugin, and 3D reconstructed images were obtained. The ICM of the blastocyst was manually identified according to morphology and colored in magenta, and the TE was colored in blue.

### Analysis of the orientation of the E–Ab axis of the blastocyst relative to the M–AM axis of the uterus

The 3D reconstructed images were used to determine the orientation of the blastocyst. We determined the centroid of the ICM and TE from a 3D reconstructed image after the volume of the ICM and TE was estimated from the segmented image of the embryo using the freehand tools in Fiji. And the E–Ab axis of the blastocyst was defined as the line connecting the centroid of the ICM and the TE. The angle between the E–Ab axis and the M–AM axis of the uterus was quantified in the range 0°–180°.

### Quantification of the blastocyst morphology in the uterus

To measure the morphology of the blastocyst in the transverse and lateral views, the lengths of the major and minor axes of the blastocyst were measured in the projected 2D images from the 3D reconstructed image of each embryo (Supplementary Figure S1B). The length of the major and minor axes of the blastocyst was measured using a freehand selection tool in Fiji. Quantification was performed in transverse and lateral views. The aspect ratio (AR) was calculated from the length of the major and minor axes. The volume and surface area of the blastocyst were calculated by multiplying the thickness of the tissue section (5 µm) by the area and perimeter, respectively.

The attachment of the blastocyst to the LE was determined according to the space between the TE of the blastocyst and the surface of the LE in the hematoxylin and eosin–stained sections, the gap between the blastocyst and the LE of more than 1 µm was defined as the space.

### Immunohistochemistry

Uteri were dissected from mice and fixed with 4% PFA in PBS at 4°C overnight. After fixation, the uteri were washed three times with PBS and incubated in 15% sucrose at 4°C for 6 h. Then, the uteri were incubated in 30% sucrose at 4°C overnight and embedded in OCT compound (No. 4583, Sakura-Finetek). The frozen block was kept in a −80°C freezer until used. For immunohistochemistry, 12–20-μm-thick frozen sections were cut with a cryostat (CM3050S, Leica), washed with PBS containing 0.1% Triton X-100 (PBST), and then incubated with Blocking One (03953–95, Nacalai Tesque) at RT for 1 h to block non-specific binding. Then, the sections were incubated with primary antibodies in Blocking One at 4°C overnight. After washing with PBST, the sections were incubated with secondary antibodies at RT for 2 h or at 4°C overnight. After washing, the samples were mounted with Fluoromount-G (0100–01, Southern Biotech).

Fluorescence images were captured using a Nikon A1 Confocal Laser Scanning Microscope (RRID:SCR_020318) or a Nikon ECLIPSE Ti2 inverted microscope (RRID:SCR_021068) equipped with a spinning disc confocal scanner unit (CSU-W1, Yokogawa) and an S-CMOS camera (ORCA-Fusion BT, Hamamatsu Photonics).

Primary antibody against Ptgs2 was purchased (Cayman Chemical Cat# 160126, RRID:AB_10079419, 1:200). Anti–E-cadherin (ECCD2)-producing hybridoma was a gift from Masatoshi Takeichi, RIKEN BDR, Japan. Alexa Fluor–conjugated secondary antibodies (Thermo Fisher Scientific; 1:500), Hoechst33258 (Molecular Probes, H3569, 1:100,000), and phalloidin (Invitrogen, A12381, 1:500) were purchased.

### Quantification of the implantation chamber depth

The depth of the IC was defined as the difference in LE length along the M–AM at the implantation site and a region 200 µm away from the implantation site in direction of the z section (Supplementary Figure S3A, B). The LE length along the M–AM axis was measured using serial transverse sections of the uterus (Supplementary Figure S3C).

### Transplantation of agarose beads into the pseudopregnant uterus

Pseudopregnant female mice were prepared by mating with vasectomized males. Con A–coated agarose beads (17-0440-03, GE Healthcare) and uncoated agarose beads (4B200, Sigma-Aldrich) were washed three times with PBS, and 10–15 beads were transplanted into the pseudopregnant uterus at 3.5 days after mating with a vasectomized male. The uteri were dissected at 4.5 days and fixed in 4% PFA. To visualize the morphology of the entire LE, the uteri were cleared with a CUBIC series (CUBIC-L: 10% N-Butyldiethanolamine and 10% Triton X-100 diluted in water; CUBIC-R+: 45% Antipyrine, 30% N-Methylnicotinamide, and N-Butyldiethanolamine diluted in water) (Susaki et al., 2020) and observed by light sheet microscopy (ZEISS, light sheet 7 (RRID:SCR_024448) or light-sheet Z.1 (RRID:SCR_020919)). The detailed morphology around the beads was analyzed using uterine sections stained with hematoxylin and eosin or immunostaining.

## Results

### The embryonic axis aligns with the uterine axis after E4.0

**FIGURE 1:**
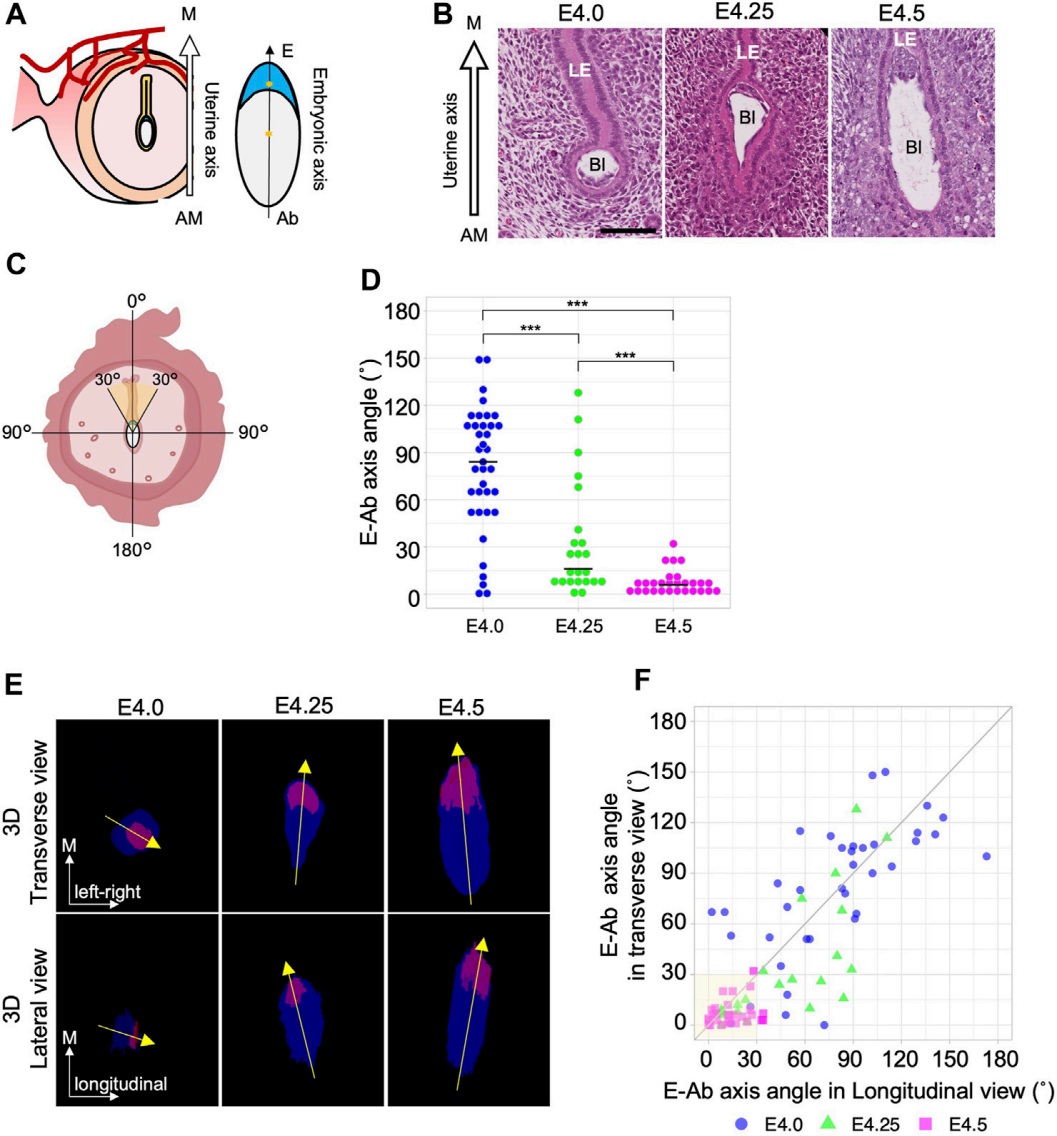
The embryonic E–Ab axis is aligned along the uterine M–AM axis during implantation. **(A)** Schematic of the embryonic axis (Embryonic–Abembryonic; E–Ab) and the uterine axis (Mesometrial–Antimesometrial; M–AM) in the transverse section of the uterus. The E-Ab axis is shown by the line connecting the centroid of the ICM and TE (asterisk). **(B)** Hematoxylin and eosin–stained images of the blastocyst in the uterus in the transverse section. The mesometrial side of the uterus is shown at the top. Bl: Blastocyst, LE: Luminal epithelium. Scale bar: 100 µm. **(C)** Schematic of the measurement of the angle between the embryonic axis and the uterine axis. **(D)** The graph shows the orientation of the embryonic axis measured using transverse sections. At E4.0, n = 37; at E4.25, n = 23; at E4.5, n = 28. **(E)** The top images show a transverse view, and the bottom images show a lateral view (image rotated 90°) of the 3D reconstructed images. The ICM and TE are colored in magenta and blue, respectively. **(F)** Scatter plot of the E–Ab axis angle in the transverse and lateral views. The gray line indicates the y = x function.

We first determined the exact time point at which the embryonic axis is aligned with the uterine axis during embryo implantation in mice. Uterine samples from four pregnant mice at each stage were obtained at different stages from E4.0 to E4.5 at 6 h intervals, and the morphology of the embryo and uterus was examined using hematoxylin and eosin–stained transverse sections. The uterine axis (M–AM axis) was defined as the axis connecting the anti-mesometrial end to the mesometrial end of the uterus (Figure 1A). The embryonic axis (E–Ab axis) of the blastocyst was defined as an axis connecting the embryonic pole where the ICM is located and its abembryonic pole opposite the ICM. We measured the angle (0°–180°) between the uterine M–AM axis and the embryonic E–Ab axis in each blastocyst (Figures 1B–D). At E4.5, the angle between the uterine and embryonic axes was <30° in every blastocyst except one (Figure 1D; n = 28; mean = 12.5°, sd = 7.7), indicating that the ICM of the blastocyst was oriented toward the uterine mesometrial side. The angle was variable at earlier stages (Figure 1B, D). A wide distribution of angles was observed at E4.0 (n = 37; mean = 79.8°, sd = 39.5), but the distribution became narrower and closer to 0° at E4.25 (n = 23; mean = 32.9°, sd = 36.3). The different trends observed in individual females may be due to differences in the timing of copulation.

To further examine the process of axis alignment, the embryonic axis orientation was observed in three dimensions by reconstructing 3D images of blastocysts from serial sections of the whole uterus (Supplementary Movie S1, S2). The 3D images of the blastocyst observed in transverse and lateral view (Supplementary Figure S1B). Representative images of transverse and lateral views of reconstructed blastocysts and the orientation of the embryonic axis are shown in Figure 1E. The angles between the uterine M–AM axis and the embryonic E–Ab axis were measured in the transverse and lateral views (Figure 1E, F). We considered the E–Ab axis to be aligned with the uterine axis when the angle was <30° in both transverse and lateral views (Figure 1C). At E4.0, the E–Ab axis was oriented in various directions, and its relationship to the uterine M–AM axis was not consistent. In 5.4% (2/37) of blastocysts at E4.0, the E–Ab axis was aligned with the uterine M–AM axis. At E4.25, 42% (10/23) of the blastocysts showed the E–Ab axis aligned with the uterine M–AM axis. There were different trends between individual females, consistent with observations in transverse sections. Two of the four females showed a variable relationship, whereas in the other two females, the E-Ab axis of all blastocysts was aligned with the M–AM axis (Supplementary Figure S2). The angles relative to the M–AM axis differed between the transverse and lateral views at E4.25. In 22% (5/23) of the blastocysts, the angle was <30° in the transverse view but >30° in the lateral view. There were no blastocysts with an angle <30° in the lateral view and >30° in the transverse view. At E4.5, the E–Ab axis of 86% (24/28) of blastocysts was aligned with the uterine M–AM axis. The variation in the angle remained relatively larger in the lateral view than in the transverse view (Figure 1F). These observations suggest that the alignment between the E–Ab axis and the uterine M–AM axis occurred between E4.0 and E4.5, and the blastocyst initially lies along the longitudinal luminal space but later becomes upright along the M–AM axis.

### Blastocysts elongate during the alignment of the embryonic and uterine axes

**FIGURE 2:**
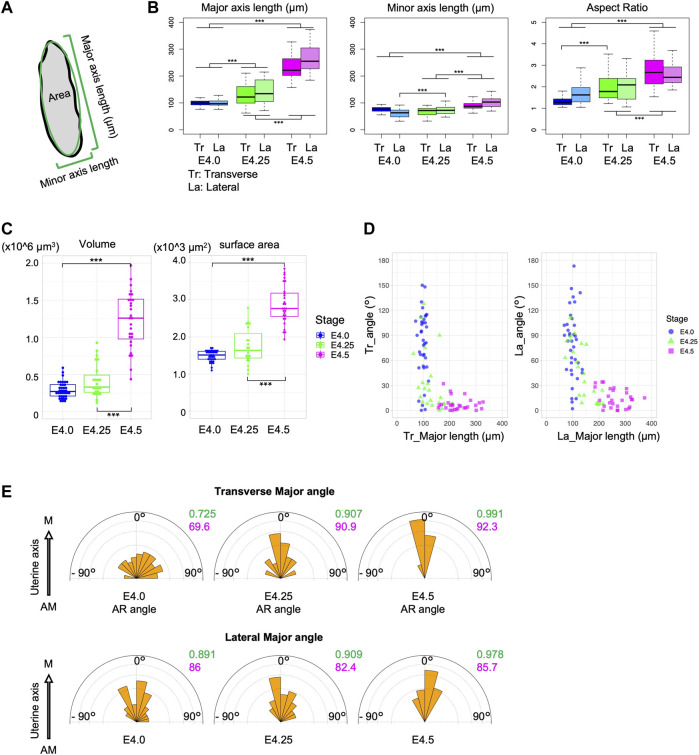
The blastocysts elongate during implantation. **(A)** Schematic of the measurement of the embryonic major and minor axes and the area in section. **(B)** Major and minor axes lengths and aspect ratio (AR) of the blastocyst in transverse (Tr) and lateral (La) views. **(C)** Volume and surface area of the blastocysts. **(D)** Correlation between the major axis length and the embryonic angle against the uterine axis in transverse and lateral views. **(E)** Angle of the major axis relative to the uterine M–AM axis. The variance and mean are shown in green and red, respectively.

At E4.5 when the axes of the uterus and the embryos are aligned, the blastocysts are significantly elongated (Figure 1B, E). We therefore hypothesized that changes in blastocyst morphology may be involved in the alignment of the embryonic and uterine axes. We then examined the relationship between blastocyst morphology and the orientation of its axis in the uterus (Figure 2A). The length of the major and minor axes, and the aspect ratio (AR) of the blastocysts were measured in both transverse and lateral views using projection images of the z section (Supplementary Figure S1B). Both the length of the major and minor axis was significantly longer at E4.5 than at E4.0 although the change in minor axis length was small. And the AR was significantly larger at E4.5 (Figure 2B). In addition, both the volume of blastocysts enclosed by the surrounding TE and the surface area of the blastocysts increased significantly from E4.0 to E4.5 (Figure 2C).

Analysis of the relationship between the major axis length of blastocysts and the orientation of the E–Ab axis showed that the E–Ab axis tended to be aligned with the uterine axis in blastocysts with a large major axis length, and this was obvious at E4.25 (Figure 2D). The major axis of the blastocyst was aligned with the uterine M–AM axis (Figure 2E). These observations suggest that the embryo is a spherical shape before the embryonic axis aligns with the uterine axis, and the blastocysts elongate along the E–Ab axis during the alignment of the embryonic axis with the uterine axis.

### Blastocyst attachment to the luminal epithelium occurs before the alignment of the embryonic and uterine axes

To determine whether the embryonic side of blastocysts is oriented toward the mesometrial side of the uterus during blastocyst attachment to the uterine epithelium, it is necessary to determine the timing of blastocyst attachment to the LE (summarized in Supplementary Table S1) and the site of blastocyst attachment to the LE. We examined the luminal space around the blastocyst and the attachment site within each blastocyst using longitudinal uterine sections from each of the four stages, E3.75, E4.0, E4.25, and E4.5 (Supplementary Table S1).

**FIGURE 3:**
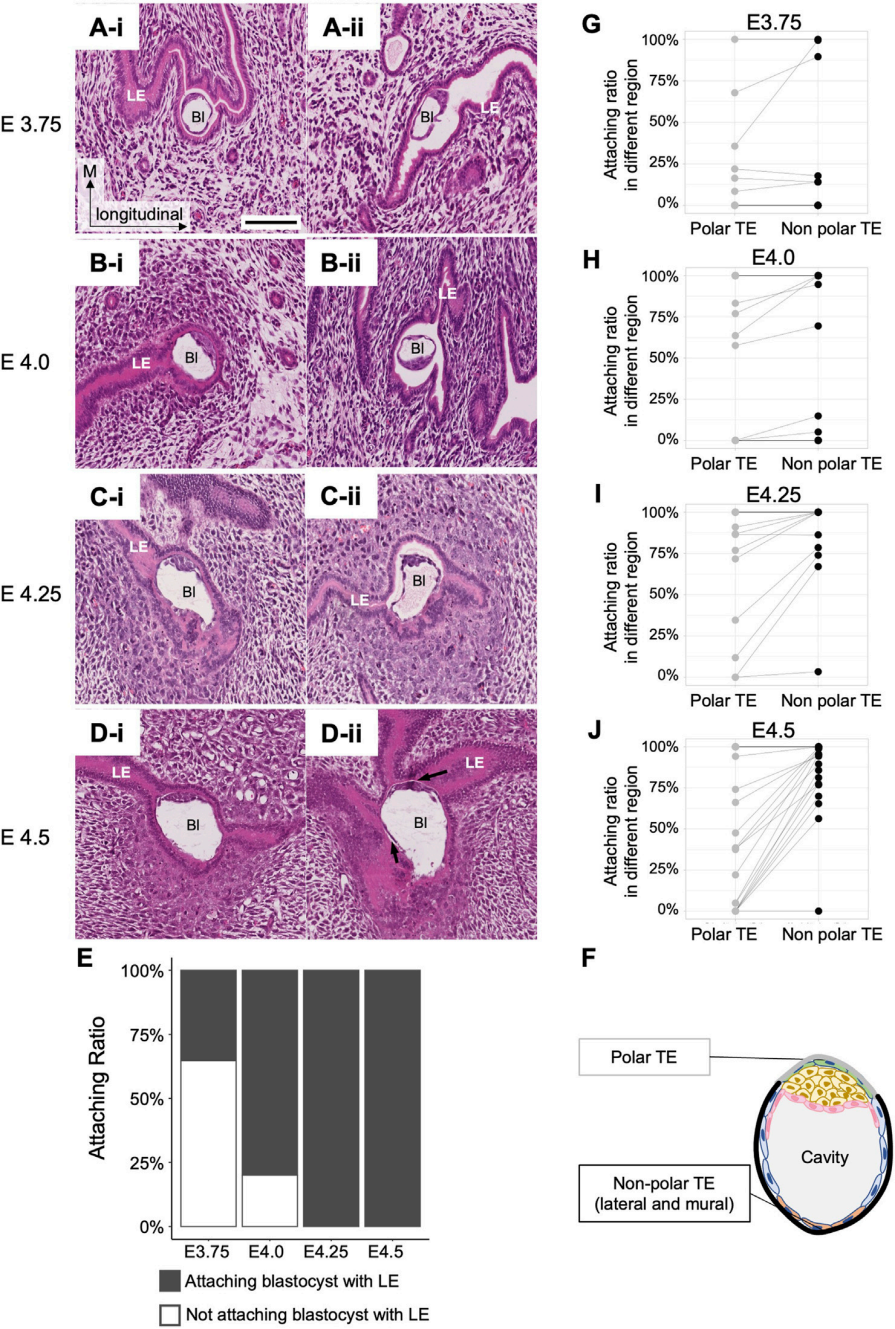
Attachment between the blastocyst and the luminal epithelium. **(A–D)** Longitudinal sections of the blastocysts in the uterus at E3.75, E4.0, E4.25, and E4.5 stained with hematoxylin and eosin. Bl: Blastocyst, LE: Luminal epithelium. The black arrows in **D-ii** indicate a small space between the blastocyst and the LE. Scale bar: 100 µm. **(E)** Rate of blastocyst attachment to the luminal epithelium. At E3.75, n = 17; at E4.0, n = 54; at E4.25, n = 22; at E4.5, n = 30. **(F)** Classification of TE regions. Polar and non-polar TE (including mural and lateral regions) were defined for the blastocyst attachment region. **(G–J)** Differences in attachment depend on the regions between the polar and non-polar TE. Each dot connected by a line represents the same blastocyst. The lines indicate the same blastocyst.

At E3.75, the lumen was partially closed, although luminal space was detected in all uteri; the variation in the size of the luminal space depended on the region where the blastocyst was located even within the same uterus. A fraction of 65% of blastocysts (11/17) was not attached to the LE (Figure 3Ai, E). Of the remaining blastocysts that were attached to the LE, three were attached to the LE only at the lateral side (Figure 3Aii), two were attached *via* the entire blastocyst perimeter, and one was attached to the LE at the mural or lateral TE (Non-polar TE). At E4.0, 80% of the blastocysts (43/54) were attached to the LE (Figure 3E); 11% (6/54) were attached to the LE partly, whereas in 69% (37/54) of the blastocysts, all the surfaces were surrounded by the LE (Figure 3Bi). The blastocysts that were not attached to the LE (11/54) were derived from the same mother (Figure 3Bii; Supplementary Table S1). At E4.25, all blastocysts were attached to the LE (Figure 3E); in 60% (13/22), the entire surface of the blastocyst was attached to the LE (Figure 3Ci), whereas in 36% (8/22), only part of the surface was attached (Figure 3Cii). At E4.5, all blastocysts (n = 30) were attached to the LE (Figure 3E); 57% (17/30) showed the entire blastocyst surface surrounded by the LE (Figure 3Di), whereas a small space between the TE and the LE was observed in 40% (12/30) of blastocysts (Figure 3Dii). One blastocyst that was not attached to the LE was thinner than the others and was considered to be developmentally delayed. Blastocysts were divided into two groups according to the attachment region: blastocysts attached to the LE at the polar TE region and those attached at the non-polar TE (lateral or mural TE) regions (Figure 3F). There were no embryos attached to the LE only at the polar side, and a large region tended to be in contact with the LE except at the polar side after E4.0 (Figures 3G–J).

**FIGURE 4:**
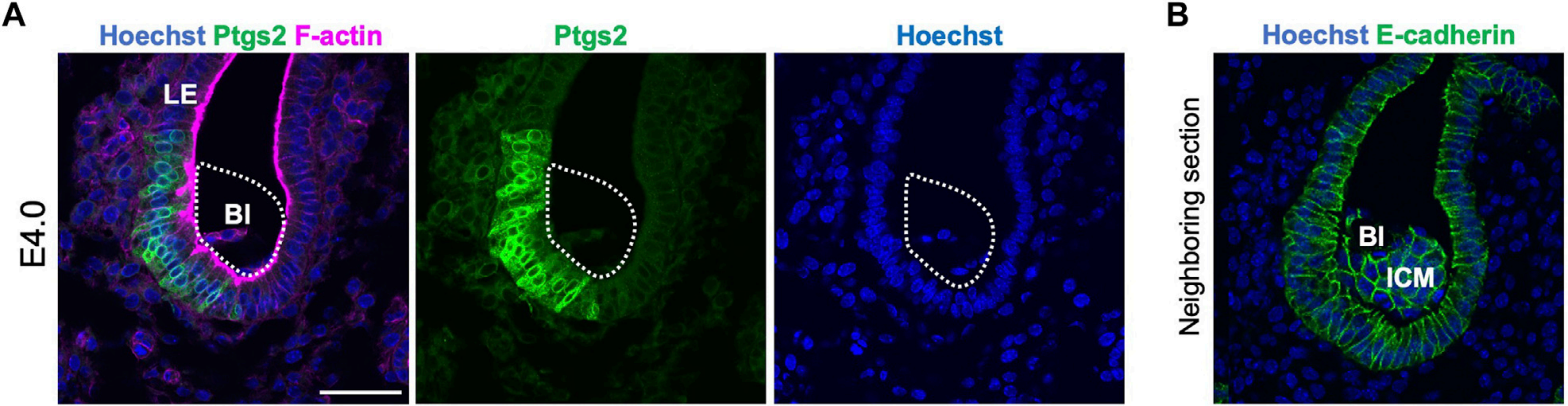
Ptgs2 expression in the luminal epithelium at E4.0. **(A)** Immunofluorescence detection of Ptgs2 in the blastocyst in the E4.0 uterus. Ptgs2 was expressed in the LE attached to the mural and lateral regions of the blastocyst. Blue, green, and magenta signals represent Hoechst, Ptgs2, and F-actin, respectively. **(B)** Neighboring section of **(A)**. Stained by Hoechst and E-cadherin. Blue and green signal represent Hoechst and E-cadherin, respectively.

We also examined the expression of markers to confirm the attachment. Prostaglandin–endoperoxide synthase 2 (Ptgs2) is expressed in the LE specifically at the site of blastocyst attachment (Chakraborty et al., 1996). At E4.0, Ptgs2 expression was detected at the site of LE contacted with the mural TE (Figure 4), supporting the histological observation of asymmetric attachment of the blastocyst, and suggesting that the LE recognized the orientation of the blastocyst at E4.0.

These observations indicate that attachment of the blastocyst to the LE begins between E3.75 and E4.0, and blastocysts are enclosed by the LE at E4.0–E4.5. Therefore, the embryonic axis is not aligned with the uterine axis at the time of attachment of the blastocyst to the LE.

### The blastocyst elongates spontaneously in the absence of luminal closure

**FIGURE 5:**
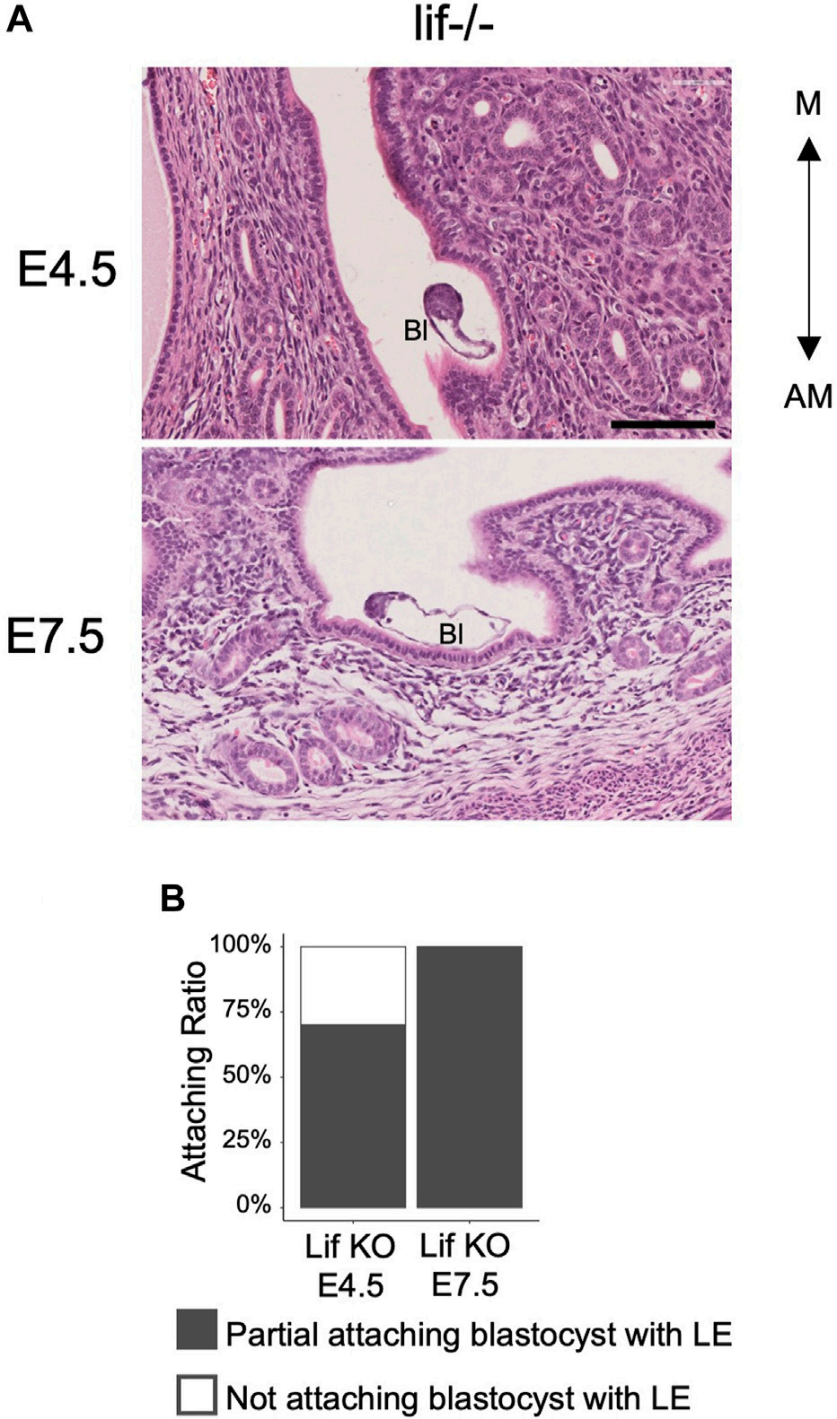
Morphology of the blastocysts and uterus in the Lif-deficient uterus. **(A)** Hematoxylin and eosin–stained images of the Lif-deficient uterus at E4.5 and E7.5 in transverse section. The blastocyst shows an oval and elongated shape. Bl: Blastocyst, Scale bar: 100 µm. **(B)** Rate of blastocyst attachment to the luminal epithelium. At E4.5, n = 10; at E7.5, n = 6.

To determine whether morphological changes of the blastocyst are caused by constriction forces generated by the LE after its narrowing and closure, embryonic diapause or the delayed blastocyst condition (Dey et al., 2004; Fenelon and Renfree, 2018) was examined. Embryonic diapause can be induced and maintained by removing the ovaries of female mice or by injecting estrogen receptor inhibitors (Yoshinaga and Adams, 1966; Lufkin et al., 2023). Leukemia Inhibitory Factor (Lif) is functionally related to the uterine receptivity of blastocysts, and in the absence of uterine Lif, blastocysts do not attach firmly to the LE, but survive in the uterus under conditions similar to embryonic diapause (Chen et al., 2000). In the pregnant Lif-deficient uterus, luminal closure was not observed at 4.5 and 7.5 days after crossing females with wild-type males. In the Lif-deficient uterus, 70% of blastocysts (n = 10) at E4.5% and 100% of blastocysts (n = 6) at E7.5 were attached partially to the LE. The luminal space around all the embryos was wide and evident (Figure 5). However, the 9 blastocysts at E4.5 and 6 blastocyst blastocysts at E7.5 in Lif-deficient uterus were elongated and the ICM was located at the tip of the blastocysts with an ellipsoidal shape. These observations suggest that the shape of blastocysts spontaneously changed to ellipsoidal even without constriction from the uterine LE.

### Implantation chamber formation is accompanied by changes in embryonic orientation

**FIGURE 6:**
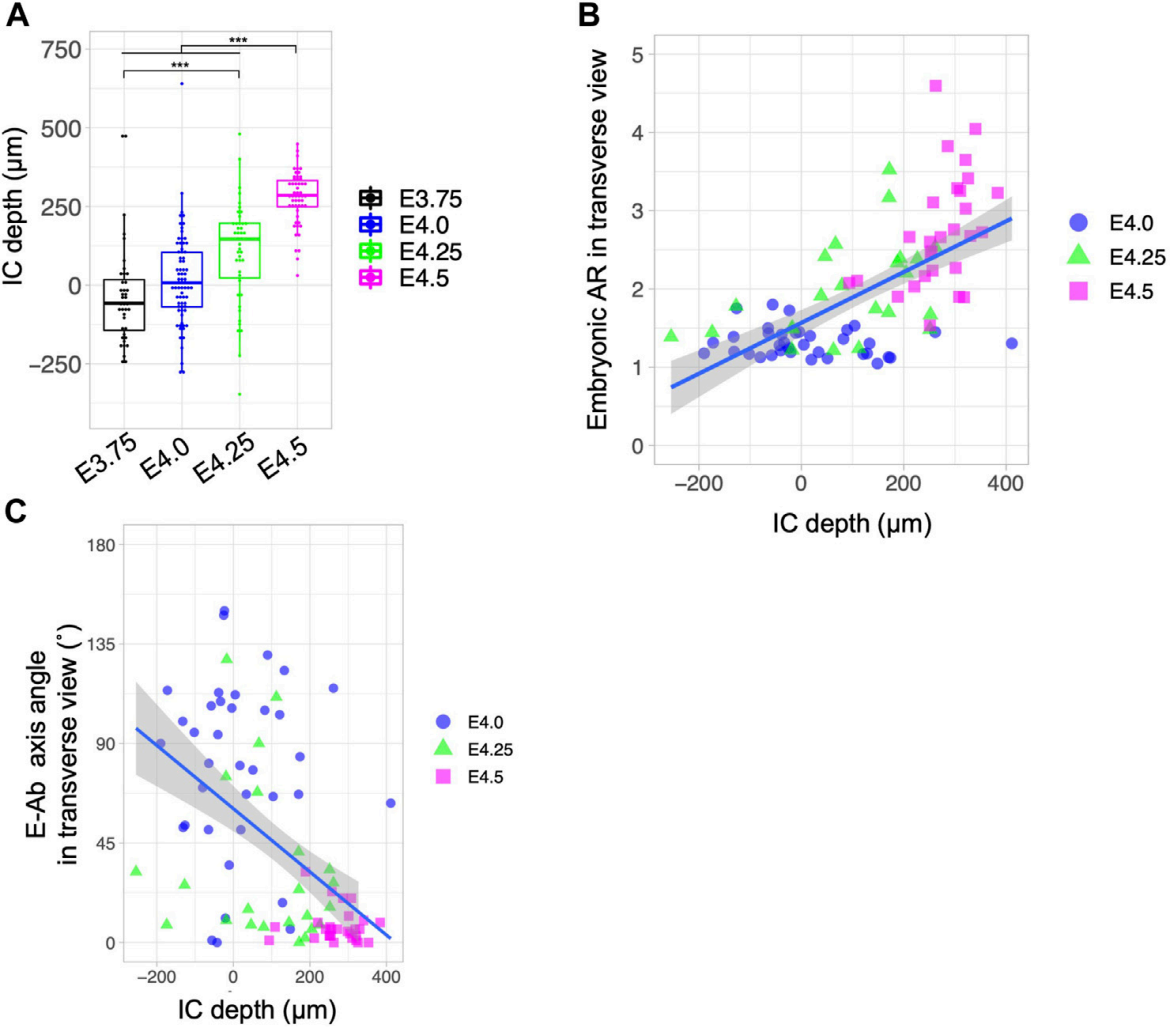
Relationship between the depth of the implantation chamber and embryonic geometry. **(A)** Depth of the implantation chamber (IC) in different developmental stages. The depth of the IC was defined as the difference between the M–AM length of the LE at the implantation site and at 200 µm away from the implantation site. **(B)** Relationship between the blastocyst AR and IC depth. The blue line shows the regression line. Pearson’s correlation coefficient was 0.639. **(C)** Relationship between the blastocyst E–Ab axis angle and IC depth. The blue line shows the regression line. Pearson’s correlation coefficient was −0.509.

When implantation is complete, the implantation chamber (IC), namely, the protrusion of the LE sheet toward the AM side specifically around the implanting embryos, is formed in the rodent uterus (Supplementary Figure S3A, B) (Enders, 1975; Madhavan et al., 2022). We investigated the relationship between the depth of the IC and the morphology and orientation of blastocysts during the process of implantation in wild-type uteri. IC depth was defined as the difference in LE length along the M–AM at the implantation site (IS) and a region 200 µm away from the implantation site (AIS) using transverse serial sections of the uterus (Supplementary Figure S3C). The IC depth increased as the embryonic stage progressed, reaching 250–300 μm at E4.5 (Figure 6A). There was a mild correlation between the embryonic AR and IC depth (Pearson correlation coefficient = 0.639; Figure 6B). Analysis of the relationship between the embryonic axis orientation and IC depth showed that the fraction of embryos in which the E–Ab axis was aligned with the uterine M–AM axis was greater at an IC depth >200 µm (Pearson correlation coefficient = −0.509; Figure 5C). These results indicate that the IC formation is accompanied by the embryo elongation and alignment of the uterus and embryonic axes.

### Concanavalin A–coated beads form arrays that align in the M–AM direction in the implantation chamber

**FIGURE 7:**
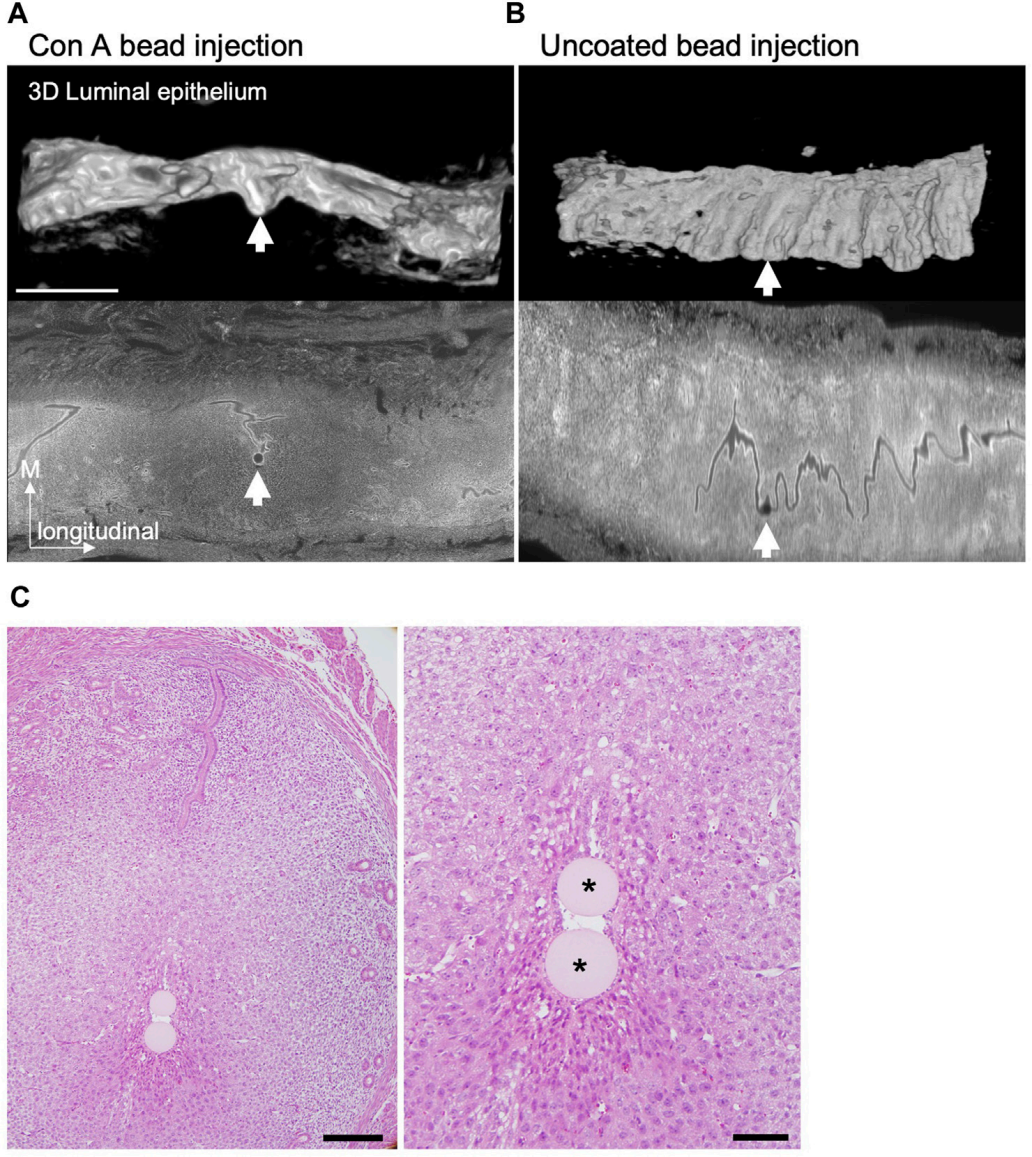
IC formation after Concanavalin A-coated beads transplantation. **(A and B)** The 3D reconstructed images of the LE at E4.5 after injection of Concanavalin A (Con A)–coated **(A)** or uncoated **(B)** agarose beads into the pseudopregnant uterus at 3.5 days were acquired by light-sheet microscopy after tissue clearing. The lower panels show the optical section of the uterus stained with propidium iodide detected by light-sheet microscopy. Scale bar: 1 mm. **(C)** Example of a case with two Con A–coated beads located in the same IC. Hematoxylin and eosin–stained images of the uterus at E4.5 after transplantation of Con A–coated beads at 3.5 days. Scale bar: 200 µm (left image), 50 µm (magnified image). Asterisks indicate the Con A–coated beads.

Finally, we investigated whether the elongated embryos actively changed orientation along the uterine M–AM axis. To test this possibility, we used Concanavalin A (Con A)–coated agarose gel beads instead of embryos for transplantation. Con A induces a decidual reaction when injected into the uterus of pseudopregnant mice expressing Ptgs2 (Supplementary Figure S4) (Wu and Gu, 1981; Herington et al., 2009). We tested whether IC formation was induced by injection of Con A–coated beads into the pseudopregnant uterus at 3.5 days after mating with a vasectomized male. IC formation was examined by light-sheet microscopy after visualization of nuclei in whole-mount uteri with propidium iodide and clearing. We found that injection of Con A-coated beads induced IC formation in the uterus similar to that occurring in normal pregnancy (Figure 7A). Transplantation of uncoated beads did not induce IC formation (Figure 7B). A total of 21 ICs were induced by Con A beads, and 5 ICs contained two or more beads. In these 5 ICs, two Con A–coated beads in close proximity formed an array surrounded by the LE in the same IC. These two-bead arrays were always aligned along the uterine M–AM axis (Figure 7C). These data suggest that IC formation can be induced by such materials as Con A–coated beads instead of actual embryos which can actively change the shape, and there is a mechanism to determine the direction in which the beads are aligned when two beads are in close proximity to each other.

## Discussion

In rodents, the placenta forms from the polar TE cells covering the ICM of the blastocyst (Rasweiler and Badwaik, 1999; Wilkinson et al., 2021). The alignment between the axis of the embryo and the axis of the uterus occurs when implantation is complete, and the ICM of the blastocyst is on the mesometrial (M) side at the post-implantation period. The alignment of the embryonic E–Ab and uterine M–AM axes, and the potential underlying mechanisms are discussed by Kirby, Potts, and Wilson (Kirby et al., 1967) and others. Unanswered questions include whether the ICM already faces the M side when the embryo first attaches to the LE, whether the ICM is passively pushed to the M side, whether the ICM actively rotates within the blastocyst, or whether the entire blastocyst is rotated by the LE or myometrium.

In this study, we investigated the timing and mechanism by which the blastocyst is oriented to align with the M–AM axis of the uterus during implantation in mice. For this purpose, we analyzed the morphological features of the embryo and the uterus in 3D during the implantation process by sampling every 6 h and performing quantitative analysis.

### The embryonic axis is not aligned with the uterine axis at the time of embryo attachment

At E3.75 and E4.0, when embryo spacing was complete (Flores et al., 2020), the embryonic axis was oriented in various directions in the uterus. From E3.75 to E4.0, the luminal space between the embryo and the uterus decreased, indicating that the embryos began to attach to the LE. The attachment site in the embryo was on the mural or lateral TE, and not on the polar TE. Ptgs2 expression in the LE was specifically detected around the embryo attachment site at E4.0, suggesting that part of the embryo was attached to the LE. Thus, at the time of embryo attachment to the LE, the embryonic and uterine axes are not yet aligned.

### Embryo elongation may contribute to the alignment of the axes of the embryo and the uterus

By the time the IC was formed at E4.5, the space in the uterine lumen was closed and the embryo was surrounded by the LE. At E4.25, some embryos showed alignment of the axis with the uterine axis, and these embryos tended to elongate. It is known that the shape of the blastocyst before hatching is affected by the ellipsoidal shape of the zona pellucida (Motosugi et al., 2005; Kurotaki et al., 2007; Honda et al., 2008). The shape of the zona pellucida was elongated, and the AR was around 1.3 around E3.5. This was far less than the AR seen at E4.5, which was higher than 2.0. In addition, the E4.5 embryos are usually hatched from the zona pellucida. These suggest that the embryo elongation during implantation was not depending on the shape of the zona pellucida. This raises the hypothesis that the embryos elongate and align along the M-AM axis as they are passively compressed by the LE rather than the zona pellucida. However, the blastocysts in the Lif-deficient uterus or those under diapause induction show elongated shape even under conditions in which the wide luminal space is maintained (Figure 5) (Kamemizu and Fujimori, 2019). This suggests that blastocysts elongate autonomously even without compression by the LE. The ICM was also located on the edge of the long-axis side of the elongated embryo, suggesting that the positioning of the ICM does not depend on external pressure on the blastocyst. However, the mechanisms by which the blastocyst elongates and the ICM localizes to the edge of the elongated blastocyst remain unclear.

We found that the orientation of the embryonic axis tended to shift toward the longitudinal axis of the uterus as the embryo began to elongate. The E–Ab axis was slightly tilted toward the longitudinal axis when viewed from the lateral side of the uterus even at E4.5 when the IC was formed (Figure 2). These findings suggest that autonomously elongated embryos tend to reside in luminal regions that allow expansion, and the embryonic axis tends to be oriented toward the M side when the IC is extended toward the anti-mesometrial (AM) side and became deeper (Figure 3). A similar phenomenon was observed in response to Con A–coated bead transplantation. Arrays formed by pairs of beads were aligned along the M–AM axis in the same IC-like structure, and Ptgs2 was expressed around the beads at the AM end of the LE. Transplantation of materials in an ellipsoidal shape, such as Con A-Coated ellipsoidal agarose bead, capable of inducing decidualization and IC formation may help determine whether the major axis of the material aligns with the M–AM axis of the uterus in the IC.

It is likely that embryos elongate independently of external compression from the uterus, and that the shape of the embryo plays a role in orienting the embryonic axis in the lumen.

### Why does the embryonic side of elongated embryos face the M side?

Given that the embryonic side containing the ICM and the opposite abembryonic side of an elongated embryo are morphologically equivalent, it remains unclear how the embryonic side is always oriented toward the M side of the uterus. One possible explanation is that the region with the ICM and the opposite side have different degrees of stiffness and are deformed in a different manner in response to compression. For example, as shown in Figure 1B, at E4.25, the side without the ICM adopts a pointed shape, whereas the side with the ICM adopts a large curvature. The ICM is a mass of cells; as such, it may show more resistance against deformation than the cavity of the blastocyst in the closing lumen from the AM side, with the embryos being oriented in a physically stable direction. It is also possible that the ICM rotates toward the M side within the blastocyst, and when the LE closes, the compression could indirectly shift the ICM toward the M side.

Another possibility is that the relative positions of the blastocyst and the LE are maintained after attachment, and the LE that is attached to the mural TE forms a protrusion at the AM side that develops into the IC. The characteristics of the mural and polar TEs at E4.5 are different at the time of implantation, and the mural TE shows a higher ability to invade the LE (Nakamura et al., 2015; Suzuki et al., 2022). Consistently when the embryo attaches to the LE before IC formation at E4.0, Ptgs2 expression is induced only in the LE region where the mural TE attached. Ptgs2 is a marker of the decidual reaction and is expressed at the stage of IC formation in most AM ends of the LE (Chakraborty et al., 1996). If the mural TE maintains contact with the LE and the part of the LE that expresses Ptgs2 protrudes toward the AM side, the mural side of the blastocyst will coincide with the AM end of the IC. As the blastocyst elongates, the abembryonic side will be at the AM end, and the embryonic side will be on the M side in the uterine lumen as a consequence. Thus, the LE changes morphology to form the IC, resulting in the alignment of the blastocyst axis with the axis of the uterus. This hypothesis is supported by observations in a mutant in which the IC develops in an oblique orientation, with the ICM located opposite the distal end of the IC (Madhavan et al., 2022).

**FIGURE 8:**
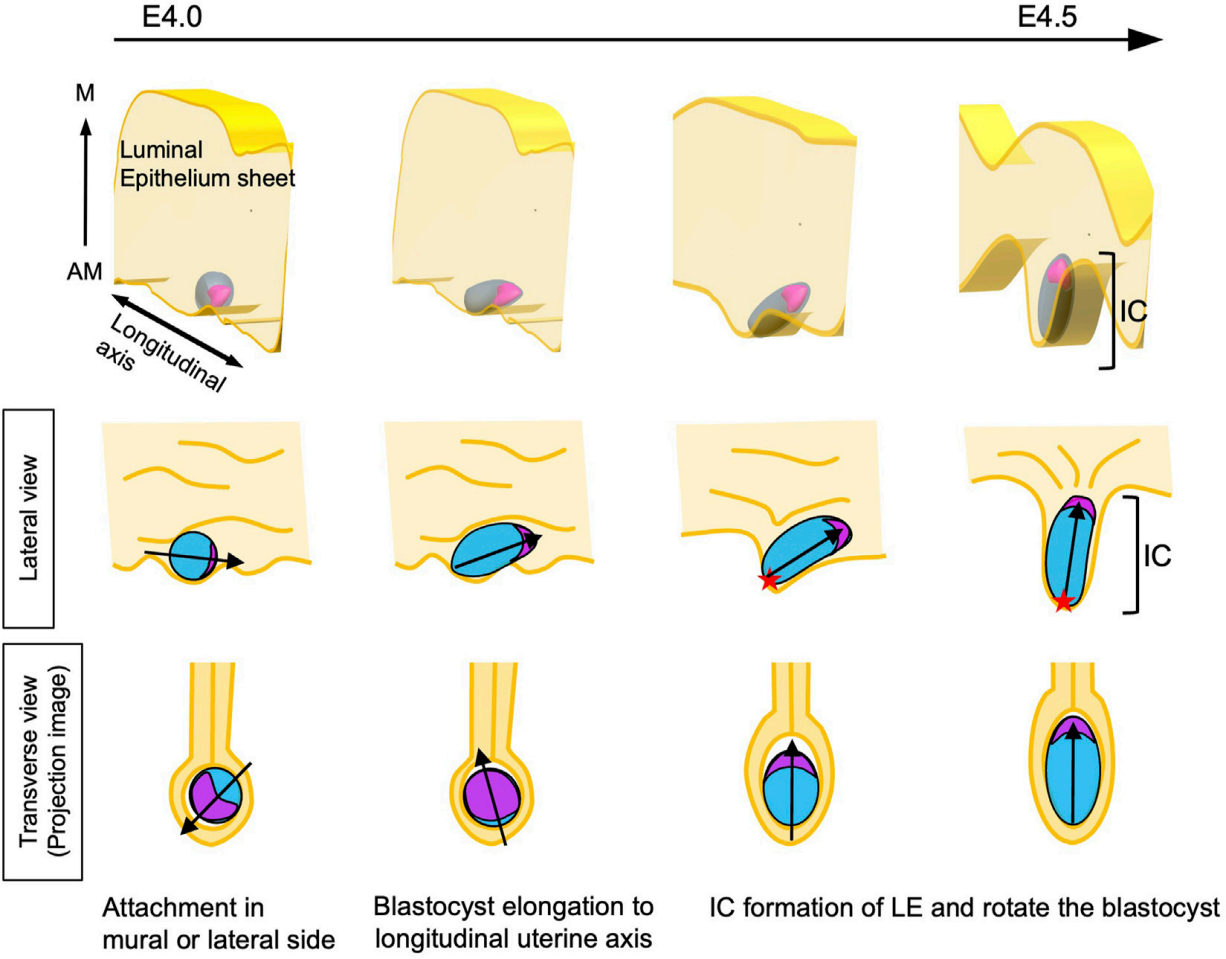
Model of the alignment of the E-Ab and M-AM axes The blastocyst attaches to the LE at the mural or lateral TE. At this point, the orientation of the blastocyst relative to the uterine M–AM axis is random. The blastocyst begins to elongate and is oriented toward the longitudinal axis of the uterus. Meanwhile, the decidual reaction is induced in LE cells attached to the mural side of the blastocyst. The LE-expressing decidual markers extend in the anti-mesometrial direction, resulting in the formation of the implantation chamber (IC). As the mural TE region moves along with LE cells expressing decidual markers, the mural TE localizes to the AM side in the uterus. The lumen is closed during IC formation, and the embryonic side of the elongated blastocyst is oriented toward the M side of the uterus, with the embryonic axis aligning with the uterine M–AM axis. The red star shows the mural TE.

The model proposed in this study is shown in Figure 8. After the embryos are distributed in the uterus and positioned on the AM side of the LE, each blastocyst initiates contact with the LE through the mural side; the decidual reaction is thus induced with Ptgs2 expression specifically at the region where mural TE attached. The embryo and the LE remain in contact, and an epithelial cell sheet of the LE extends toward the AM side to form the IC. Meanwhile, the blastocyst elongates autonomously. As the LE attached to the mural TE extends to the AM side to form the IC, the elongated blastocyst rotates to orient the ICM toward the M side, resulting in the alignment of the embryonic and uterine axes. Several epithelial growth factors (EGFs) and Integrins have been reported as molecules required for implantation (Fritz et al., 2014; Aplin and Ruane, 2017). While the heparin-binding EGF-like growth factor (HB-EGF) has been suggested as the earliest molecule that mediates communication between the embryo and LE at the attachment (Das et al., 1994) and ItgB1 may play a role in the invasion of mTE into LE (Wang et al., 2002; Bondarenko et al., 2023), we need to examine the exact timing of their expression and function for further study. And many questions remain unanswered, such as the molecular basis of the attachment, the molecular mechanisms underlying the exchange of information between the blastocyst and the LE, and why the IC specifically protrudes toward the AM side.

## Data Availability

The raw data supporting the conclusion of this article will be made available by the authors, without undue reservation.

## Supplementary material

SUPPLEMENTARY MOVIE S1 Movies of reconstructed blastocysts. The movies show serial images of the blastocysts in Figure 1B. Scale bar: 100 µm.

SUPPLEMENTARY MOVIE S2 Movies of reconstructed blastocysts. Rotated 3D images of the segmented blastocysts shown in Figure 1E. The ICM and TE are colored in magenta and blue, respectively. Scale bar: 100 µm.

SUPPLEMENTARY FIGURE S1 The plane of the uterus and blastocyst in observation. **(A)** Schematic of the uterus. Three axes of the uterus; M-AM axis, longitudinal axis, and left-right axis. **(B)** Schematic of the blastocyst. The morphology of the blastocyst was measured in two planes; in the transverse and the longitudinal planes of the uterus. The projected images were used, and indicated as transverse view and lateral view.

SUPPLEMENTARY FIGURE S2 Variation in the orientation of the embryonic axis relative to the uterine axis among individual females. Scatter plot of the E–Ab axis angles in transverse and lateral views at E4.0, E4.25, and E4.5; related to Figure 1. The gray line indicates the y = x function.

SUPPLEMENTARY FIGURE S3 The implantation chamber formation during implantation. Schematic of the implantation chamber (IC) formation. The LE surrounding the blastocyst protrudes toward the AM side of the uterus. **(A)** The LE sheet was a flattened board like shape regardless of the location of the embryo before IC formation. **(B)** The LE sheet surrounding the embryo protruded toward the AM side of the uterus during implantation. **(C)** The length of the LE was measured using the transverse section of the uterus along the M–AM axis. The difference in LE length at 200 μm away from the IS and at the implantation site is defined as the IC depth.

SUPPLEMENTARY FIGURE S4 Concanavalin A–coated beads induced the expression of Ptgs2 in surrounding tissues. Immunostaining of Ptgs2 around the Con A–coated beads; related to Figure 7. The Ptgs2 signal was detected on the LE and stromal cells around the bead. E-cadherin was expressed in LE cells.

SUPPLEMENTARY TABLE S1 Comparison of the blastocyst attachment rates in individual females.
