# Supplementary material for: Effects of blastocyst elongation and implantation chamber formation on the alignment of the embryonic axis and uterine axis in mice
Source: Front Cell Dev Biol. 2024 Jun 14;12:1421222. doi: 10.3389/fcell.2024.1421222 (PMC11211524; doi:10.3389/fcell.2024.1421222)
Supplement: Supplementary file 3 [file DataSheet1.docx]

Supplementary Material

**Blastocyst elongation and implantation chamber formation coordinate the alignment of the embryonic and uterine axes in mice**

**Jun Sakurai, Sanae Oka, Yoko Higuchi, Sonoko Ohsawa and Toshihiko Fujimori***

*Corresponding author. E-mail: [fujimori@nibb.ac.jp](mailto:fujimori@nibb.ac.jp)

# Supplementary Figures and Tables

## Supplementary Figures


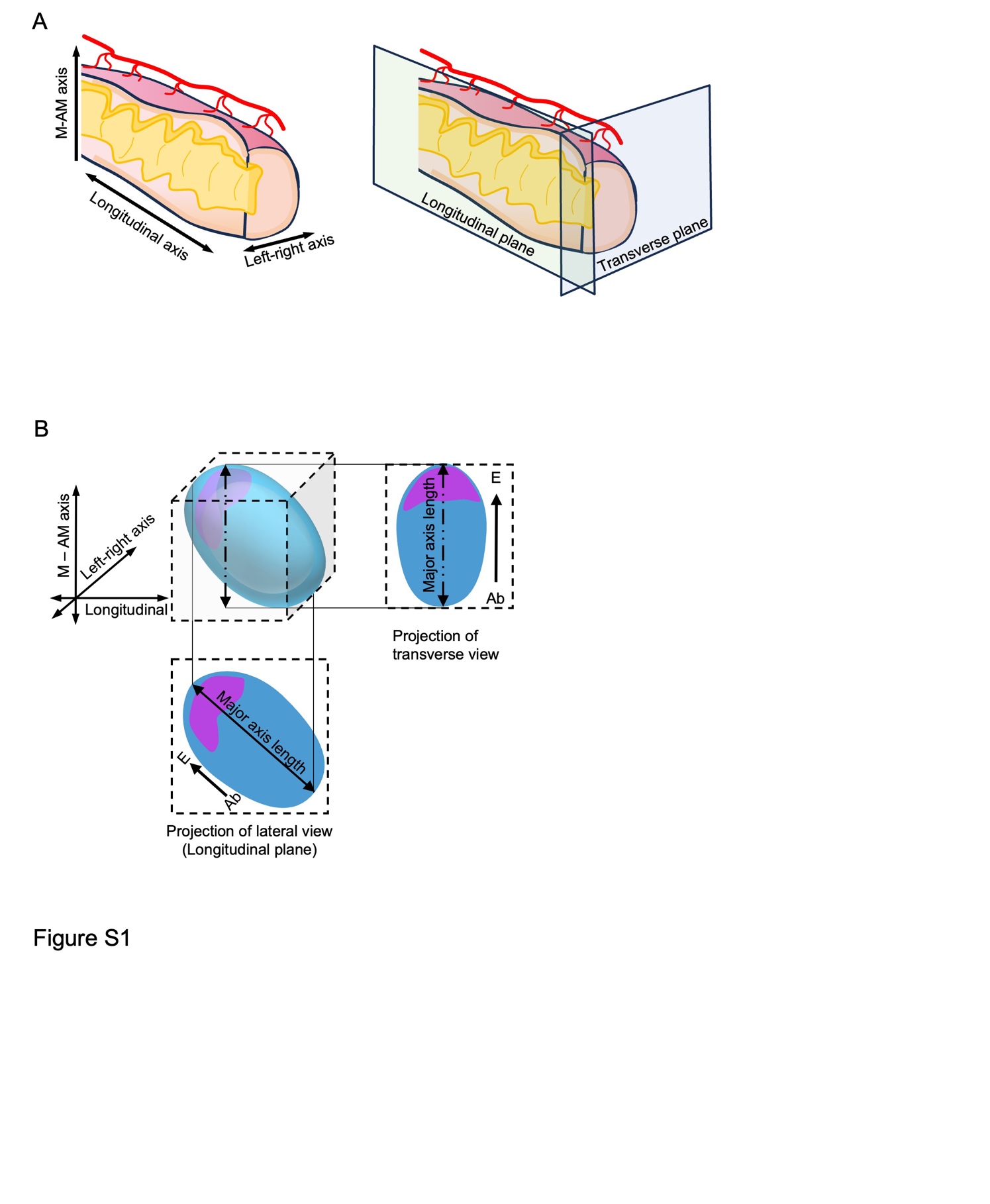


Figure S1. The plane of the uterus and blastocyst in observation

(A) Schematic of the uterus. Three axes of the uterus; M-AM axis, longitudinal axis, and left-right axis. (B) Schematic of the blastocyst. The morphology of the blastocyst was measured in two planes; in the transverse and the longitudinal planes of the uterus. The projected images were used, and indicated as transverse view and lateral view.

**Figure S2. Variation in the orientation of the embryonic axis relative to the uterine axis among individual females.**

Scatter plot of the E–Ab axis angles in transverse and lateral views at E4.0, E4.25, and E4.5; related to Figure 1. The gray line indicates the y=x function.


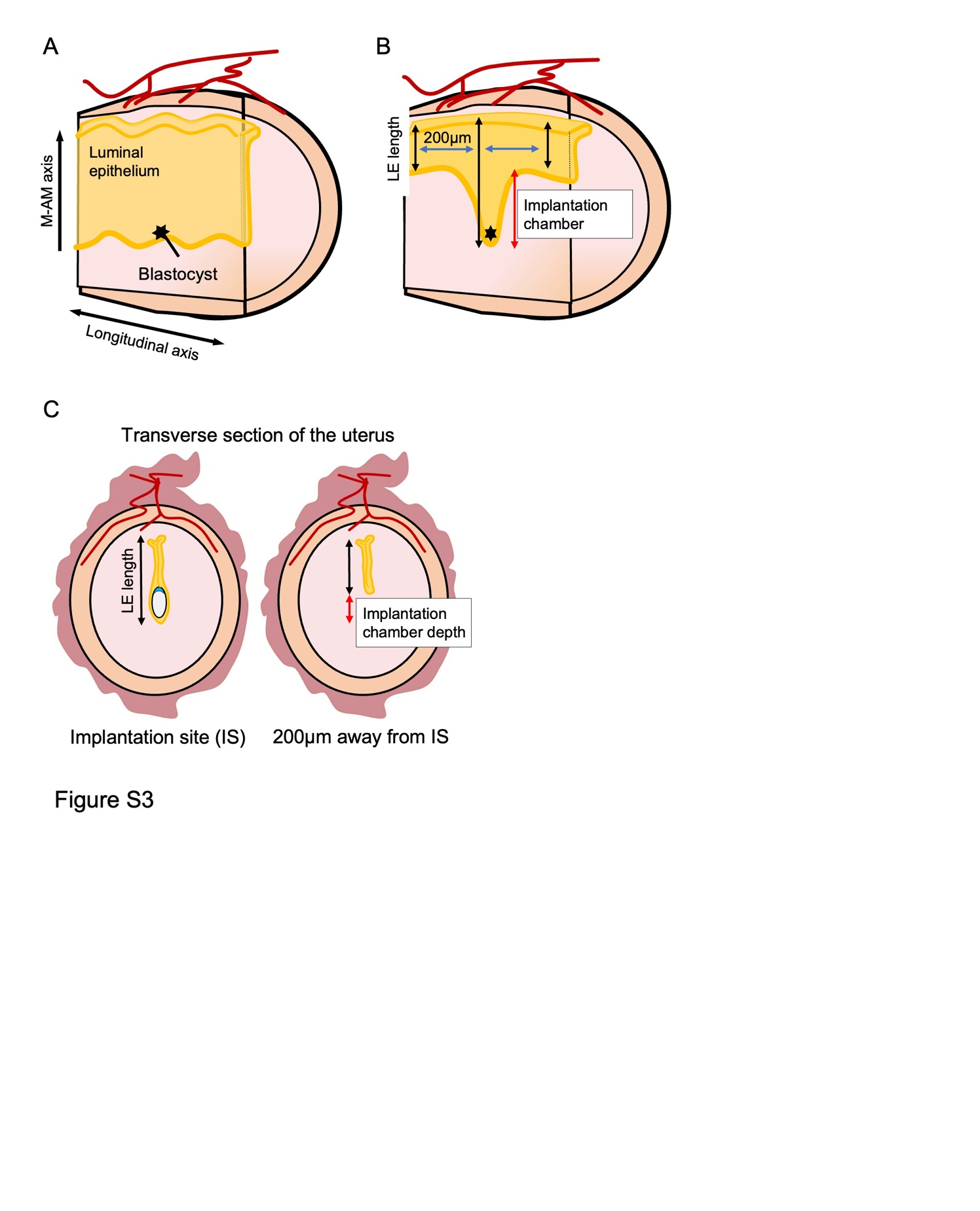


Figure S3. The implantation chamber formation during implantation

Schematic of the implantation chamber (IC) formation. The LE surrounding the blastocyst protrudes toward the AM side of the uterus. (A) The LE sheet was a flattened board like shape regardless of the location of the embryo before IC formation. (B) The LE sheet surrounding the embryo protruded toward the AM side of the uterus during implantation. (C) The length of the LE was measured using the transverse section of the uterus along the M–AM axis. The difference in LE length at 200um away from the IS and at the implantation site is defined as the IC depth.

**Figure S4.** **Concanavalin A–coated beads induced the expression of Ptgs2 in surrounding tissues.**

Immunostaining of Ptgs2 around the Con A–coated beads; related to Figure 7. The Ptgs2 signal was detected on the LE and stromal cells around the bead. E-cadherin was expressed in LE cells.

## Supplementary Table

**Table 1.** **Comparison of the blastocyst attachment rates in individual females.**

| Stage | Female# | No. of blastocysts | Attached to LE | Attach entire region of the blastocyst | Attach partial region of the blastocyst | No Attach to LE |
| --- | --- | --- | --- | --- | --- | --- |
| E3.75 | #1 | 9 | 6 | 2 | 4 | 3 |
|  | #2 | 8 | 0 | 0 | 0 | 8 |
| E4.0 | #1 | 12 | 1 | 0 | 1 | 11 |
|  | #2 | 14 | 14 | 14 | 0 | 0 |
|  | #3 | 13 | 13 | 9 | 4 | 0 |
|  | #4 | 15 | 15 | 14 | 1 | 0 |
| E4.25 | #1 | 9 | 9 | 4 | 5 | 0 |
|  | #2 | 13 | 13 | 9 | 4 | 0 |
| E4.5 | #1 | 5 | 5 | 3 | 2 | 0 |
|  | #2 | 12 | 11 | 6 | 5 | 1 |
|  | #3 | 13 | 13 | 8 | 5 | 0 |
